# Parallel Multi-Scale Semantic-Depth Interactive Fusion Network for Depth Estimation

**DOI:** 10.3390/jimaging11070218

**Published:** 2025-07-01

**Authors:** Chenchen Fu, Sujunjie Sun, Ning Wei, Vincent Chau, Xueyong Xu, Weiwei Wu

**Affiliations:** 1 Department of Computer Science and Engineering, Southeast University, Nanjing 210000, China; chenchen_fu@seu.edu.cn (C.F.); sjjsun@seu.edu.cn (S.S.); 220191687@seu.edu.cn (N.W.); weiweiwu@seu.edu.cn (W.W.); 2 North Information Control Research Academy Group Co., Ltd., Nanjing 210000, China

**Keywords:** depth estimation, semantic segmentation, multi-task learning, metric learning

## Abstract

Self-supervised depth estimation from monocular image sequences provides depth information without costly sensors like LiDAR, offering significant value for autonomous driving. Although self-supervised algorithms can reduce the dependence on labeled data, the performance is still affected by scene occlusions, lighting differences, and sparse textures. Existing methods do not consider the enhancement and interaction fusion of features. In this paper, we propose a novel parallel multi-scale semantic-depth interactive fusion network. First, we adopt a multi-stage feature attention network for feature extraction, and a parallel semantic-depth interactive fusion module is introduced to refine edges. Furthermore, we also employ a metric loss based on semantic edges to take full advantage of semantic geometric information. Our network is trained and evaluated on KITTI datasets. The experimental results show that the methods achieve satisfactory performance compared to other existing methods.

## 1. Introduction

With the technological breakthroughs in the field of computer vision, research fields such as autonomous driving and 3D reconstruction have also developed rapidly. These fields inevitably apply visual technology to perceive three-dimensional space to assist decision making. The application of 3D vision adds depth information to 2D, paying more attention to information recognition and substance understanding in a 3D space. In addition to directly using sensors to obtain the information on depth, attempts have been made to obtain 3D data directly from 2D data. Depth estimation is widely applied in various fields, playing a key role in robot navigation and autonomous driving scene reconstruction.

Derived from the multi-view stereo geometry theory, the principle of depth estimation is closely related to the natural biological binocular vision system. Early depth estimation methods mainly relied on matching pixels from images obtained from multiple and calculating the depth of pixels through triangular matching of feature points. With the rapid development of deep learning, people have proposed methods based on deep learning. Through continuous training on datasets, deep neural networks can learn the relationship between images and depth. Depth estimation methods based on deep learning are divided into two categories—supervised learning and self-supervised learning. Supervised learning methods [1,2,3] require a large number of manually annotated depth image pairs as training data, whose generalization ability is particularly limited by the size and diversity of the dataset. According to the theory of structure from motion, the idea of self-supervised learning to estimate the depth and the pose from the video sequence has received attention in scientific research [4,5]. Image re-projection is performed using the estimated depth and pose. Then the photometric loss between the re-projected image and the target image provides a supervisory signal for the training of the neural network.

In recent years, many new methods have emerged in the field of self-supervised monocular depth estimation, and the results of models have been improved on Zhou’s baseline [4]. These methods [5,6] mainly rely on pixel loss and smoothness loss, resulting in the loss of weakly textured regions. What is more, tiny objects in the scene cannot be correctly estimated. Godard et al. [6] introduced a minimum re-projection loss and auto uncertainty masking technique to improve training accuracy. Although many studies on self-supervised monocular depth estimation come out one after the other, the accuracy of the results is still affected by dynamic objects, scene occlusions, lighting differences, and sparse textures.

The combination of semantic information and depth information can be used as supplementary knowledge of 3D space, thereby improving the performance of depth estimation. Considering the relevance of semantic segmentation and depth estimation tasks [7,8], we propose a novel multi-task self-supervised monocular video depth estimation pipeline. Taking unlabeled video sequences and the semantic labels generated by a pre-trained model as input, we design a parallel multi-scale semantic-depth interactive fusion network. The features of two tasks at different scales can achieve continuous transmission and parallel interaction, which improves the accuracy of depth estimation.

Although the complementary information in the feature space of different tasks enables the network to autonomously emphasize feature information, the implicit feature interaction fusion method may introduce some feature noise. Therefore, we actively mine the information of edges in semantic images and add a metric loss based on semantic edges to the photometric loss to take full advantage of semantic geometric information. Metric samples are obtained according to semantic boundaries, and the strategy of boundary sample classification based on the reconstruction of semantic images of adjacent frames is designed to increase the robustness of the metric loss.

Models trained on our pipeline outperform most other recent works. The main innovations of the paper are summarized as follows.

- A scheme that combines semantic segmentation to estimate depth is proposed to implement an end-to-end pipeline. We adopt a multi-stage feature attention network (MSFAN) for the feature extraction of RGB images instead of the traditional U-net structure encoder to improve the accuracy of the depth estimation task.
- We introduce a semantic segmentation task by sharing feature extraction network MSFAN, and add a parallel semantic depth interactive fusion module (PSDIFM) to achieve the bidirectional complementarity of feature information between different tasks.
- The total multi-task loss function is designed to adapt to the new pipeline, and the metric loss based on semantic edges is added to refine the depth of the edges, promoting the further improvement of depth estimation results.
- Our network pipeline is trained on KITTI dataset and evaluated. The results of KITTI and Make3D datasets show that the network pipeline designed by us achieves satisfactory performance compared to other existing methods.

## 2. Related Work

### 2.1. Depth Estimation

Before the invention of neural networks, stereo algorithms were used for depth estimation of multiple images. After that, Eigen et al. [1] applied neural networks to introduce depth estimation from a single image by training the network on sparse labels provided by LiDAR scans. This work was refined in the following studies by improving training techniques and frameworks. With the application of fully convolutional neural networks, Laina et al. [9] proposed a fully convolutional network based on residual structure and used a pre-trained encoder for feature extraction, which improved the resolution of results. Mancini et al. [10] used fully convolutional networks and optical flow labels to detect the obstacle. However, existing approaches still suffer from limitations in sensor data quality, insufficient cross-scene generalization, and challenges in modeling complex dynamic environments.

### 2.2. Self-Supervised Depth Estimation

The bottlenecks of supervised methods are poor generalization performance and difficulty in obtaining ground truth of depth. On the other hand, stereo depth estimation relies on camera extrinsic parameters and complex networks, thereby, self-supervised monocular depth estimation is beginning to be studied. Zhou et al. [4] first proposed a monocular-trained framework combined by a depth network and a pose network that trained on sequences of video frames. Aiming to solve the problems of moving objects and occlusion, Godard et al. [6] designed an auto masking and brought out Monodepth2 framework, which has been one of the most frequently used baselines. Yang et al. [11] introduced a normal vector of surface and consistency constraints of the normal vector of depth to improve the performance. Mahjourian et al. [5] extracted the information on adjacent frames and proposed to use the ICP loss to make the depth results in adjacent frames have stronger consistency. For the uncertainty of depth estimation, Poggi et al. [12] designed a novel method. The uncertainty is estimated by image flipping and integrated, and the statistical mean and variance in the image are estimated according to different models. Many thoughts have been carried out on previous works according to the model architecture [13] and loss functions [14,15] and have achieved significant improvement. Despite these advances, challenges persist in handling intricate motion patterns, robustness to abrupt illumination changes, and the inability to recover absolute metric scale.

### 2.3. Semantic-Guided Depth Estimation

Semantic segmentation [16,17] is to assign a predefined semantic label to each pixel in the image. Fully convolutional and encoder–decoder structures are used for feature extraction and refinement segmentation, resulting in significantly improved segmentation performance. Due to the similarity of depth estimation and semantic segmentation tasks, semantic information has been introduced to improve depth estimation performance. A scheme to handle moving dynamic objects to avoid miscalculation of photometric losses is proposed in [8]. Chen et al. [18] designed a multi-task framework with shared encoders to perform semantic segmentation and depth estimation with a consistent structure. Choi et al. [19] designed different fusion modules between decoder network layers. Zhu et al. [20] proposed a measure of boundary consistency between segmentation and depth, and Jung et al. [21] designed a semantics-guided metric loss. They explicitly guide the training of depth edges with semantic edges to provide a new signal for optimizing depth results. However, these methods do not fully utilize the feature information in encoder and decoder networks, resulting in the loss of information during network transmission. To make up for that, the pipeline we designed takes into account the parallel interaction of different scale features in different tasks. Inspired by Jung et al. [21], the metric loss based on the strategy of boundary sample classification is added to the photometric loss, improving the accuracy of depth estimation.

## 3. Proposed Approach

### 3.1. Problem Statement

Self-supervised monocular depth estimation computes depth values for camera images on a pixel-by-pixel basis without using any ground truth labels. Given three consecutive frames of a video sequence, they are numbered in time order as $I_{t-1}$, $I_{t}$ and $I_{t+1}$. We also refer to $I_{t}$ as the target image, $I_{t-1}$ and $I_{t+1}$ as the source images $I_{s}$. According to Monodepth2 [6], the pipeline is trained with both the depth and six degree-of-freedom relative poses of the source and target images $T_{t\to s}$. Based on the world static assumption that the view change in driving scenes is only caused by a moving camera, we can synthesize the corresponding frames of the target image using the source image and the relative poses. With known camera intrinsics *K*, we can use the following equation to calculate the projected pixel coordinates:

(1) $$p_{s}∼KT_{t\to s}{\hat{D}}_{t}\left(p_{t}\right)K^{-1}p_{t},$$

where $p_{t}$ is the homogeneous coordinates of the pixel in the target image and $p_{s}$ is the re-projected coordinates of $p_{t}$ by $T_{t\to s}$. Through bilinear sampling, the surrounding pixels of $p_{s}$ in $I_{s}$ can be obtained, and is calculated as the pixel value of the corresponding position of the synthetic target image. Based on the theory of structure from motion, the synthesized image ${\hat{I}}_{t}$ should be consistent with the original image $I_{t}$. We calculate the minimized photometric loss using the structural similarity index metric in combination with the $L_{1}$ pixel loss.

### 3.2. Network Architecture

#### 3.2.1. Pipeline

**Figure 1:**
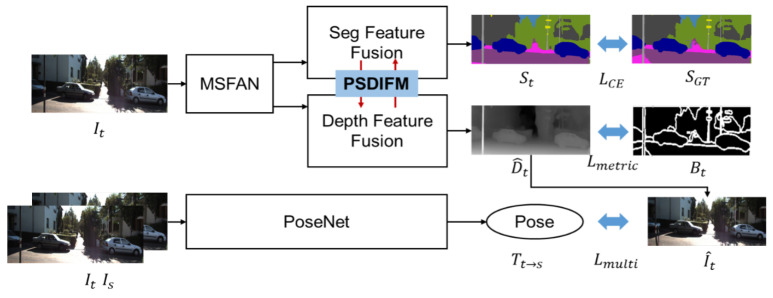
Overview of the proposed Parallel Multi-Scale Semantic-Depth Interactive Fusion Network. $I_{t}$ and $I_{s}$ represent the target and source images. MSFAN extracts multi-scale semantic and depth features, which are fused by PSDIFM into the semantic prediction $S_{t}$ and depth prediction ${\hat{D}}_{t}$. The network includes a pose estimation module (PoseNet) to compute the transformation $T_{t\to s}$ for view synthesis. $S_{GT}$ and $B_{t}$ denote the ground-truth semantic map and semantic edge boundary, respectively. Loss functions $L_{CE}$, $L_{metric}$, and $L_{multi}$ supervise the respective tasks.

The pipeline of the parallel multi-scale semantic-depth interactive fusion network we designed is shown in Figure 1. The input of the depth estimation task and semantic segmentation task is both single RGB images. Two tasks share a multi-stage feature attention network (MSFAN) and output multiple scales of features. More feature details can be preserved at each resolution, and the parameter redundancy caused by repeated calculations can be effectively avoided. In the next feature fusion process, we designed a parallel semantic-depth interactive fusion module (PSDIFM). The depth and semantic prediction tasks each uses separate network branches to fuse features at multiple scales level by level, and features of different scales are interacted and fused between tasks. At the same time, two adjacent frames of images $I_{s}$ and $I_{t}$ are input to the PoseNet to obtain a 6DoF pose for the calculation of photometric loss.

The boundary set $B_{t}$ of the image is extracted from the semantic pseudo-label, and then the metric loss $L_{metric}$ of the depth feature at the corresponding position is calculated according to the feature comparison idea in the metric learning method, which realizes the explicit guidance of the depth by the semantic boundary. The semantic segmentation task uses only the semantic pseudo-labels generated by the pre-trained model. The labels are only used to distinguish objects of different semantics and do not need to represent specific categories. This can reduce the reliance on real labels for semantic tasks and make the training data more uniform without introducing additional datasets.

#### 3.2.2. Multi-Stage Feature Attention Network

Many existing depth estimation approaches that obtain high-resolution feature maps use the method of first reducing and then increasing the resolution. The networks are usually built on ResNet to convert images into low-resolution features, similar to U-Net, DeconvNet, etc. Although low-resolution features have rich semantics, images lose spatial structure information in the process of feature extraction. In previous work, we can infer that the performance of the model on the task of depth estimation depends to some extent on the resolution of the input image, i.e., the results of high-quality images are usually better than those of low-quality ones. Inspired by dense prediction tasks such as semantic segmentation, multi-level parallel feature extraction networks can better preserve features at different resolutions. HR-Depth [22] has applied multi-level feature extraction to the network and confirmed the effectiveness of the method, but lacks the interactive fusion of different stages.

**Figure 2:**
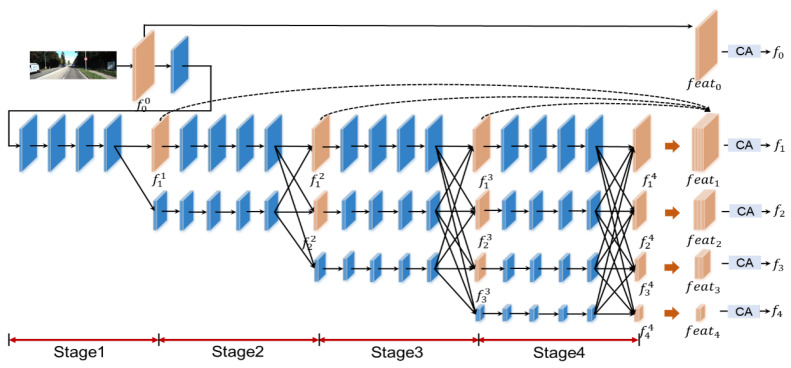
Multi-Stage Feature Attention Network (MSFAN).

HRNet [16] parallelizes feature maps of different resolutions, and adds interactions to feature maps of different resolutions in forward convolution stages. Inspired by the framework, we adopt a multi-stage feature attention network (MSFAN) instead of the classic U-Net structure including a multi-stage details enhancement module. As shown in Figure 2, the network has four data streams and four stages, each of which fuses information between different scales, while extracting the feature maps, we observed a significant improvement in other methods using ResNet as the backbone.

Feature maps are transmitted in parallel at different levels, and as the network is extended, the feature information from the previous stages cannot be effectively preserved and utilized. To enrich the feature information in the final output, the network first stitches multi-stage features together and then performs detail enhancement using the idea of an attention mechanism. Compared to traditional resolution-reducing encoders, this multi-scale parallel design enables better preservation of spatial detail, particularly near depth boundaries. However, maintaining multiple resolution streams throughout the network introduces moderate computational and memory overhead. This is a conscious design trade-off in favor of accuracy over efficiency. In Section 4.3, we provide empirical comparisons demonstrating that our method maintains competitive performance under acceptable resource usage. The traditional spatial attention and channel attention lack the consideration of the relationship between features and coordinate positions in two-dimensional images. Global pooling is used in the channel attention mechanism to encode the spatial information as channel descriptors, making it difficult to preserve location information. Unlike traditional channel attention, we adopt a coordinate attention mechanism [23] to emphasize the interdependence between location information. This is particularly useful in-depth estimation, where depth discontinuities often align with semantic or geometric boundaries. Let $f_{l}^{i}$ denote the feature map from the *i*-th stage at resolution level *l*, and ε(.) denote channel-wise concatenation. The output feature $f_{l}$ at level *l* is defined as follows:

(2) $$f_{l}=\left\{\begin{array}{cc} C(f_{l}^{0}),\; & l=0\\ C(\varepsilon \left(f_{l}^{l},f_{l}^{l+1},\ldots ,f_{l}^{4}\right)),\; & l\in \{1,2,3,4\} \end{array}\right.$$

where C(.) represents the coordinate attention module applied to the concatenated features. This allows the network to enhance important features at each resolution stage by leveraging both spatial and contextual cues.

#### 3.2.3. Parallel Semantic Depth Interactive Fusion

In multi-task training, two tasks with high similarity can be considered as the primary and secondary task, and the secondary task can provide more feature information to the primary task [24]. Features at different scales of the auxiliary task have different effects on the main task due to the different receptive field sizes of features. For example, local semantic maps can provide useful information for depth prediction and improve the depth estimation of object edges. On the other hand, local depth maps provide less information about the semantics of the scene, but when the receptive field is expanded, depth maps reveal the shapes of the object, implying semantic information about the scenes.

**Figure 3:**
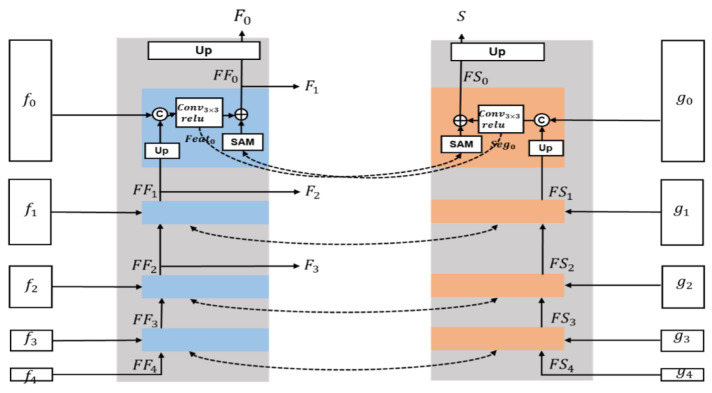
Architecture of the Parallel Semantic-Depth Interactive Fusion Module (PSDIFM). The left and right branches process multi-scale depth features $f_{i}$ and semantic features $g_{i}$, respectively. At each level, the Spatial Attention Module (SAM) enables bidirectional interaction by enhancing one branch with attention maps generated from the other. $FF_{i}$ and $FS_{i}$ denote the fused outputs at scale i. “Up” indicates bilinear up-sampling. Dashed lines represent cross-branch fusion.

We designed a parallel semantic-depth interactive fusion module, which allows the features of a task to interact between multiple scales. The interacted features can be used for feature combinations at other scales. In general, this approach can provide more adequate feature information for the training of each task and improve the network performance. In Figure 3, the parallel fusion module of depth estimation task is shown in blue and the parallel fusion module of semantic segmentation is shown in orange. They interact with features at four scales by a designed feature interactive fusion mechanism. The depth estimation task outputs four fused features $F_{0}$, $F_{1}$, $F_{2}$, and $F_{3}$, and the final predicted depth map of the network can be obtained after the convolution layer and activation function. The semantic segmentation task outputs a semantic graph *S* with the same size as the input image.

As shown in Figure 3, in the depth estimation task, the smaller-sized feature $FF_{l+1}$ is first upsampled, and is spliced with $f_{l}$, and then pass through convolutional and relu layers to obtain the fused feature $Feat_{l}$ on a single task. Similarly, in the semantic segmentation task, $FS_{l+1}$ is upsampled and spliced with $s_{l}$, and the $Seg_{l}$ feature is output after passing through convolutional and activation layers. The $Feat_{l}$ and $Seg_{l}$ can be calculated as follows:

(3) $$Feat_{l}=\left\{\begin{array}{cc} F(\varepsilon \left(U\left(FF_{l+1}\right),f_{l}\right)),\; & l\in \{0,1,2,3\}\\ f_{l},\; & l=4 \end{array}\right.$$

(4) $$Seg_{l}=\left\{\begin{array}{cc} F(\varepsilon \left(U\left(FS_{l+1}\right),f_{l}\right)),\; & l\in \{0,1,2,3\}\\ s_{l},\; & l=4 \end{array}\right.$$

where F(.) is a block consisting of convolution operation followed by an activation, U(.) is an upsampling block.

The parallel interactive fusion mechanism is designed with reference to the idea of spatial self-attention [25]. The Spatial Attention Module (SAM) can be used as a gate function to control the information flow and enable the network to refine useful information autonomously. $F_{l}$ is added with $S_{l}$ distilled by SAM in the semantic segmentation task and will be used for feature fusion in the upper layer. Meanwhile, $S_{l}$ is also distilled using SAM and added with $F_{l}$ in the semantic network.

(5) $$FF_{l}=\left\{\begin{array}{cc} Feat_{l}⊕SAM\left(Seg_{l}\right),\; & l\in \{0,1,2,3\}\\ Feat_{l},\; & l=4 \end{array}\right.$$

(6) $$FS_{l}=\left\{\begin{array}{cc} Seg_{l}⊕SAM\left(Feat_{l}\right),\; & l\in \{0,1,2,3\}\\ Seg_{l},\; & l=4 \end{array}\right.$$

The features $F_{l}$ that meet the task requirements after fusion can be calculated from $FF_{l}$, and the final output depth map can be obtained after the convolution layer and activation function.

(7) $$F_{l}=\left\{\begin{array}{cc} F(U\left(FF_{l}\right)),\; & l=0\\ FF_{l-1},\; & l\in \{1,2,3\} \end{array}\right.$$

### 3.3. Boundary Alignment Loss

One of the optimization goals of the depth estimation task is to make the edges of objects in the depth map more clear. After adding the semantic segmentation task, the correspondence between the depth map and the object edges in the semantic map can be established, so that the boundaries of depth images are aligned with semantic images. Since the pixels on the semantic boundary may have large depth differences, metric loss can be calculated inspired by recent work [21], using depth feature maps within the neighborhood locations of the semantic boundary. Based on this idea, our work designs a boundary alignment loss to further enhance the use of semantic boundaries.

When extracting boundary pixel samples, a neighborhood of size Z×Z is constructed with each pixel i on the semantic map as the center. In each neighborhood, the pixels belonging to the same semantic category as the central pixel are marked as a set of positive pixels $P_{i}^{+}$, and the pixels with different semantic categories are marked as a set of negative pixels $P_{i}^{-}$. The set of boundary pixels $B_{t}$ needs to satisfy the formula as follows:

(8) $$|count\left(P_{i}^{+}\right)-count\left(P_{i}^{-}\right)|\leq T$$

**Figure 4:**
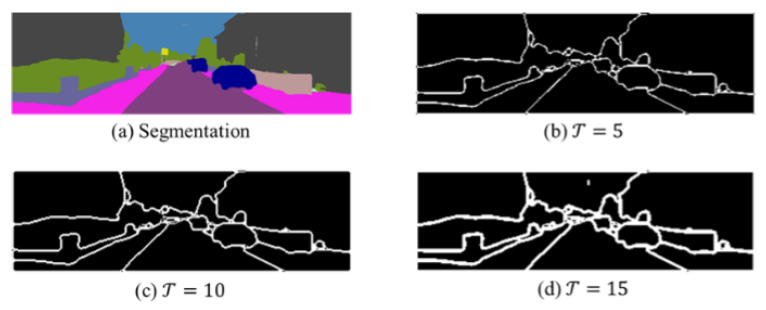
Boundary results $B_{t}$ under different threshold T.

As shown in Figure 4, both the neighborhood length *Z* and the threshold T significantly affect the extracted boundary maps. Based on visual quality and consistency with object contours, we empirically set T=10 in all experiments. This value strikes a balance between suppressing weak/noisy gradients and preserving meaningful edge structures. Further evaluation of sensitivity to T will be considered in future work.

**Figure 5:**
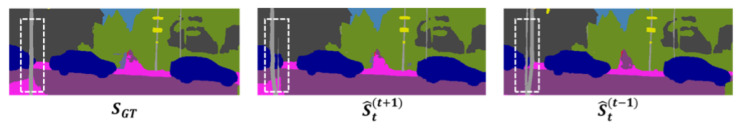
Images of semantic inconsistency.

For three consecutive images $I_{t-1}$, $I_{t}$ and $I_{t+1}$, we have corresponding semantic images $S_{t-1}$, $S_{GT}$ and $S_{t+1}$. By the method of view synthesis, we obtain the synthesized semantic maps corresponding to $I_{t}$, which are denoted as ${\hat{S}}_{t}^{\left(t-1\right)}$ and ${\hat{S}}_{t}^{\left(t+1\right)}$. As shown in Figure 5, semantic inconsistency is mainly distributed at the boundary of the image, which is caused by the depth estimation task and the pose prediction task. It can be used as a self-supervised signal to optimize the training of the whole network.

In Algorithm 1, $B_{c}$ refers to the predicted depth boundary pixels, extracted by computing gradient edges from the estimated depth map. $B_{ct}$ denotes the predicted semantic boundary pixels, computed from the semantic segmentation output. $S_{GT}$ is the semantic ground truth label map, used to identify label transitions in the neighborhood of depth edges. On the boundary sample set $B_{t}$, a classification strategy based on semantic reconstruction between frames is adopted. The set of boundary samples $B_{t}$ are divided into basic boundary set $B_{gt}$ and semantically inconsistent boundary set $B_{ct}$. A higher weighted metric loss is computed for the semantically inconsistent boundary pixels to enhance the effectiveness of the metric loss. Algorithm 1 describes the extraction process of $B_{ct}$. $B_{gt}$ is calculated by Equation (9).

(9) $$B_{gt}=B_{t}-B_{ct}$$

The feature map can be normalized as ${\hat{F}}_{l}=\frac{F_{l}}{∥F_{l}∥}$.
**Algorithm 1:** Semantic Inconsistent Boundary Pixel Extraction **Input**: Synthetic semantic maps ${\hat{S}}_{t}^{\left(t+1\right)}$ and ${\hat{S}}_{t}^{\left(t-1\right)}$, Pseudo label $S_{GT}$, Semantic boundary pixel set $B_{t}$ **Output**: Semantically inconsistent boundary pixel set $B_{ct}$
1:Initialize the set of semantically inconsistent pixels $B_{c}\leftarrow \emptyset$2:**for all** each pixel coordinates $p_{t}$ in $I_{t}$ **do**3:    Obtain pixel categories: ${\hat{S}}_{t}^{\left(t+1\right)}\left(p_{t}\right)$, ${\hat{S}}_{t}^{\left(t-1\right)}\left(p_{t}\right)$4:    Obtain pixel class $S_{GT}\left(p_{t}\right)$ on the pseudo-label map5:    **if** ${\hat{S}}_{t}^{\left(t+1\right)}\left(p_{t}\right)\neq S_{GT}\left(p_{t}\right)$ **then**6:        Add *p_t_* to *B_c_*7:    **end if**8:    **if** $p_{t}\notin B_{c}$ **and** ${\hat{S}}_{t}^{\left(t-1\right)}\left(p_{t}\right)\neq S_{GT}\left(p_{t}\right)$ **then**9:        Add *p_t_* to *B_c_*10:    **end if**11:**end for**12:$B_{ct}\leftarrow B_{c}\cap B_{t}$13:**return** *B_ct_*


We grouped the features in each patch of the depth feature map into three classes (i.e., anchor, positive, and negative) following the corresponding pixel locations in the semantic image patch. We defined positive distance $d^{+}\left(i\right)$ and negative distance $d^{-}\left(i\right)$ as the mean of the cosine distance.

(10) $$\begin{array}{cc} \; & d^{+}\left(i\right)=\frac{1}{|P^{+}|}\sum_{j\in P_{i}^{+}}\sqrt{2-2{\hat{F}}_{l}\left(i\right){\hat{F}}_{l}\left(j\right)}\\ \; & d^{-}\left(i\right)=\frac{1}{|P^{-}|}\sum_{j\in P_{i}^{-}}\sqrt{2-2{\hat{F}}_{l}\left(i\right){\hat{F}}_{l}\left(j\right)} \end{array}$$

The triplet metric loss [26] is calculated separately on the two sample point sets $B_{gt}$ and $B_{ct}$. For the points in the base boundary sample point set $B_{gt}$, the metric loss on the depth features of the *l*-th layer is as follows:

(11) $$L_{metric}^{gt}\left(l\right)=\sum_{i\in B_{gt}}\max\left(d^{+}\left(i\right)-d^{-}\left(i\right)+m,0\right)$$

Similarly, for the points in the semantically inconsistent boundary sample point set $B_{ct}$, the metric loss on the depth features of the *l*-th layer is as follows:

(12) $$L_{metric}^{ct}\left(l\right)=\sum_{i\in B_{ct}}\max\left(d^{+}\left(i\right)-d^{-}\left(i\right)+m,0\right)$$

The total boundary alignment loss can be calculated as:

(13) $$L_{metric}\left(l\right)=\frac{\gamma_{1}L_{metric}^{gt}\left(l\right)+\gamma_{2}L_{metric}^{ct}\left(l\right)}{\left|B_{gt}\right|+\left|B_{ct}\right|}$$

### 3.4. Multi-Task Loss

The network needs to calculate the loss on the depth estimation results at four different scales. From the source image $I_{s}$, the pose $T_{t\to s}$ and the depth value ${\hat{D}}_{t}$ calculated by the depth network, the synthesized target image ${\hat{I}}_{t}$ can be calculated. If the depth and pose are accurate, the images ${\hat{I}}_{t}$ and $I_{t}$ should be consistent at the pixel level, so the pixel difference between them can be used to train the network. Denoting the pixel error between images as $L_{pixel}$ we have

(14) $$L_{pixel}=\min_{I_{s}}pe\left(I_{t},{\hat{I}}_{t}\right)$$

In Equation (14), pe(.) represents the pixel photometric error consisting of $L_{1}$ loss and Structured Similarity (SSIM) loss. The $L_{1}$ loss calculates the difference in each valid pixel point. The structured similarity function measures the similarity between two images. pe(.) can be calculated as follows:

(15) $$pe=\frac{\alpha }{2}\left(1-SSIM\left(I_{t},{\hat{I}}_{t}\right)\right)+\left(1-\alpha \right){∥I_{t}-{\hat{I}}_{t}∥}_{1}$$

In real-world scenes, not all scenes conform to the static assumption that the presence of moving objects in the scene can interfere with pixel errors. We apply a stationary pixel mask [6] to filter the stationary pixels that maintain the same appearance between the front and back frames. The pixel mask $M_{pixel}$ is calculated in Equation(16).

(16) $$M_{pixel}=\min_{I_{s}}pe\left(I_{t},{\hat{I}}_{t}\right)<\min_{I_{s}}pe\left(I_{t},I_{s}\right)$$

For image $I_{t}$ and the corresponding predicted depth ${\hat{D}}_{t}$, the edge smoothing loss [6] is calculated as in Equation (17), with $\partial_{x}$ and $\partial_{y}$ denoting the horizontal and vertical gradients. $L_{smooth}$ can make the gradient of depth and RGB images consistent.

(17) $$L_{smooth}\left(I_{t},{\hat{D}}_{t}\right)=|\partial_{x}{\hat{D}}_{t}|e^{-|\partial_{x}I_{t}|}+\left|\partial_{y}{\hat{D}}_{t}\right|e^{-|\partial_{y}I_{t}|}$$

The losses on a single scale can be calculated as follows:

(18) $$L_{single}=\mu M_{pixel}⊙L_{pixel}+\lambda L_{smooth}\left(I_{t},{\hat{D}}_{t}\right)$$

In order to reduce the influence of noise on the loss calculation, it is necessary to assign different weights to different scales of losses. The total loss is calculated by Equation (19).

(19) $$L_{base}=\frac{1}{\left|Scale\right|}\sum_{l\in Scale}\left(\frac{1}{2^{l}}L_{single}^{l}\left(I_{t},{\hat{I}}_{t}\right)\right)$$

In this study, a pre-trained model is used to construct corresponding semantic labels for RGB images in video sequences, and the difference between the semantic results predicted by the semantic network branches and the pseudo-labels is calculated as the cross-entropy loss $L_{CE}=-\sum_{i=1}^{n}y_{i}\log\left({\hat{y}}_{i}\right)$ to train the network. To achieve end-to-end training, the loss of the multitask pipeline is calculated as follows:

(20) $$L_{multi}=\mu_{1}L_{base}+\mu_{2}L_{CE}+\mu_{3}L_{metric}$$

## 4. Experiments

### 4.1. Datasets and Evaluation Metrics

**KITTI.** The KITTI dataset [19] has been implemented in training and evaluation, which contains RGB images captured by cameras and real depth data captured by radar scanning equipment. Since the dataset lacks semantic labels, existing semantic model has been adopted generating labels for each image. We use the KITTI Eigen split [1] and remove all static frames that are the same with related works [4,6]. This gives rise to 39,910 samples for training and 4424 samples for validation. The test dataset has a total of 697 samples.

**Make3D.** The Make3D dataset [27] contains 400 single training RGB with depth map pairs and 134 test samples for depth estimation tasks. The RGB images have high resolution, while the depth maps are provided at low resolution by laser camera.

For semantic supervision, we use pseudo-labels generated by a DeepLabv3+ model with a ResNet-101 backbone, pre-trained on the Cityscapes dataset. Although this model is not fine-tuned on KITTI or Make3D, it produces reasonably accurate segmentation results in urban scenes. Some domain mismatches may still occur, particularly in non-driving environments like those in Make3D, leading to noisy semantic labels in certain regions. The evaluation metrics in this paper are consistent with previous works [4,6]. For mean absolute relative error (AbsRel), square relative error (SqRel), root mean square error (RMSE), and root mean square logarithmic error (RMSE_log), lower is better. For the accuracy under threshold ($\delta <1.25^{i},i=1,2,3$), higher is better. All depth images are calculated within 80 m during validation. Because the self-supervised models trained on monocular video sequences cannot recover the true depth, we use the same scale factor as Monodepth2 [6] to determine the true scale information on depth values.

### 4.2. Network and Training Details

The code implementation is based on PyTorch 2.7.1 library trained with NVIDIA 3090 GPU and Intel(R) Core(TM) i7-9700F CPU @ 3.00 GHz. The size of each input image is 192 × 640 pixels and we do random color jitter augmentation while loading the dataset. We initiate the parameters of some high-resolution representation layers with the HRNet [16] model pre-trained on Cityscapes, and the depth and segmentation layers are randomly initialized.

The network is trained for 20 epoches by the Adam optimizer and the learning rate is set to $10^{-4}$. During training, we set the batch size to 12. We set α to 0.85 in Equation (15), and the weights $\left(\mu_{1},\mu_{2},\mu_{3}\right)$ in the total loss are set to 1.0, 0.2, and 0.02 in Equation (20), respectively. These values were chosen based on empirical tuning to balance the magnitude and impact of each loss term. A larger $\mu_{2}$ or $\mu_{3}$ was found to overemphasize auxiliary tasks (semantic segmentation and edge consistency), leading to reduced depth accuracy. This choice provides a stable convergence behavior and the best validation performance.

### 4.3. Quantitative and Qualitative Results

**Figure 6:**
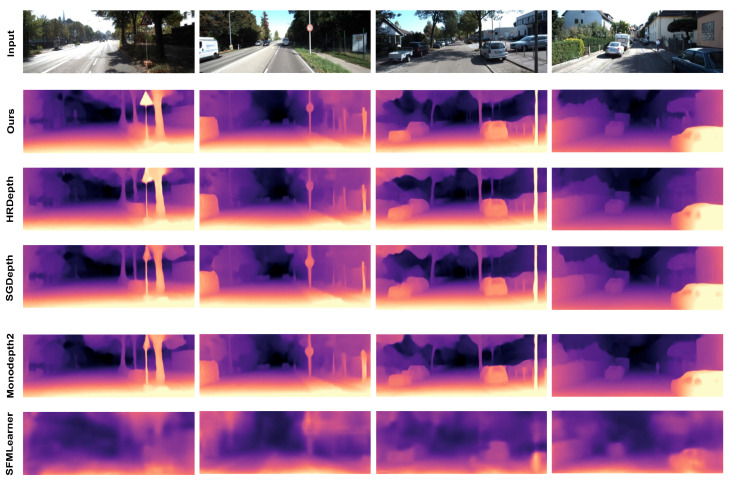
Qualitative results of depth estimation on KITTI dataset.

**Evaluation on KITTI Dataset.** We evaluated the main performance of the KITTI Eigen split and the quantitative results are shown in Table 1. We compare them to the methods that use monocular image sequences, trained with or without the segmentation task: *Y* means with segmentation and *N* means without the task. The evaluation results represent our best practice for the depth estimation task, which outperforms other self-supervised approaches. Compared with works of single task, our proposed approach shows a great improvement, which reflects the effectiveness of the multi-stage feature attention network and the guidance of segmentation task. The shared feature extraction network strengthens information through the transmission of the parallel features and attention mechanism. What is more, our work also has advantages over methods of semantic guidance [8,18,19]. Although they add semantic segmentation tasks to improve depth estimation results, they ignore the loss of information in the transmission of the network. By introducing the parallel multi-scale semantic-depth interactive fusion network, our method improves the a1 index by 0.3% over FeatDepth [28], and by 1.8% over SAFENet [29]. Through qualitative results shown in Figure 6, we compare our method with HR-Depth [22], Monodepth2 [6], and SFMLearner [4]. It can be seen that depth estimation can identify more details of distant scenes and better represent the outlines of objects, which also confirms the effectiveness of our method.

**Figure 7:**
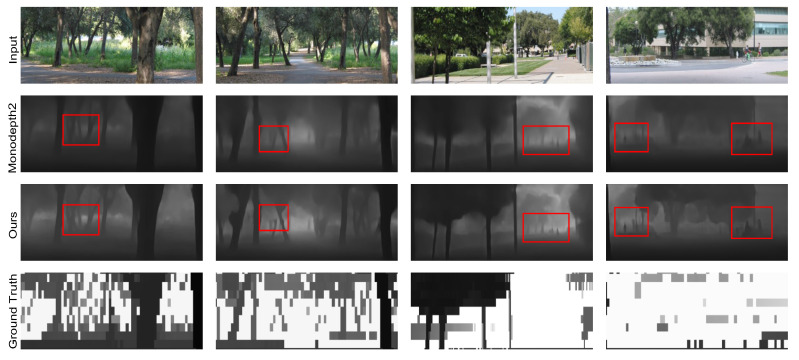
Qualitative results of depth estimation on Make3D dataset.

**Evaluation on Make3D Dataset.** To verify the generalization ability of the proposed method in training scenarios, which have never been seen before, we compare the performance of several self-supervised models on Make3D dataset. As shown in Table 2, under the same evaluation protocol as [27], our model outperforms other works and exhibits good generalization performance. The qualitative results are shown in Figure 7, which optimizes the depth estimation results in terms of details in the scenes.

Although our method is primarily designed to improve depth estimation accuracy, particularly around object boundaries, real-time inference is also important for practical applications such as autonomous driving and robot navigation, while we did not perform a full runtime benchmark, our model is built with efficiency in mind. Specifically, we adopt a lightweight HRNet backbone and streamlined parallel fusion modules to balance accuracy and computational cost. We acknowledge the importance of inference speed and will explore this aspect more thoroughly in future versions of the work.

### 4.4. Ablation Study

To determine the effectiveness of each contribution proposed in our method, we conducted an ablation study. (The ablation results are based on single-run experiments due to time and resource constraints, while consistent trends were observed across training epochs, future work will include repeated trials and statistical analysis (e.g., standard deviation or *t*-tests) to better quantify the significance of the observed improvements.) The result is shown in Table 3, starting from the baseline model and individually adding our contributions up to the full method. First, the addition of multi-stage feature attention network (MSFAN) shows an improvement. After that, introducing the semantic segmentation network without interactive fusion of features between tasks further improves the baseline. Then, applying the parallel semantic-depth interactive fusion module (PSDIFM), we achieved a greater performance than before. In the end, the metric loss is added to training. Ablation studies show that all techniques are designed to refine the depth representation. When they are combined, they can offer significant improvements to the depth estimation task.

## 5. Conclusions

In this paper, we propose a self-supervised monocular depth estimation pipeline incorporating semantic tasks. Through the designed parallel multi-scale semantic-depth interactive fusion network, our self-supervised depth estimation network could learn semantic-aware features to improve the performance of depth estimation. Furthermore, the total multi-task loss function is designed to adapt to the new pipeline, and the metric loss based on semantic edges is added to refine the depth of the edges. To prove the effectiveness of different modules in our method, various experiments were conducted. The experimental results show that our method is more effective than other state-of-the-art methods. While our approach shows strong performance, it also has limitations. In particular, the quality of semantic features may impact depth prediction, as we rely on pseudo-semantic labels generated by a pre-trained segmentation model. Although the depth decoder learns in an end-to-end manner and can tolerate some noise, robustness under imperfect or noisy semantic labels remains a challenge. In future work, we plan to investigate the effect of semantic label noise by introducing synthetic corruption or dropout during training, and evaluate model performance under these settings. Furthermore, we aim to improve the real-time performance of our model through optimization techniques such as pruning, quantization, or knowledge distillation. These approaches will help adapt our method for deployment in resource-constrained environments, such as embedded or mobile platforms used in robotics or autonomous driving.

## Figures and Tables

**Table 1:** Quantitative results of depth estimation on KITTI dataset for distance within 80 m.

Method	Sup	AbsRel ↓	SqRel ↓	RMSE ↓	RMSE_log ↓	*a*_1_ ↑	*a*_2_ ↑	*a*_3_ ↑
SFMLearner [4]	N	0.183	1.595	6.709	0.270	0.734	0.902	0.959
Vid2Depth [5]	N	0.163	1.240	6.220	0.250	0.762	0.916	0.968
GeoNet [14]	N	0.153	1.328	5.737	0.232	0.802	0.934	0.972
Casser [30]	N	0.141	1.026	5.291	0.215	0.816	0.945	0.979
CC [31]	F	0.140	1.070	5.326	0.217	0.826	0.941	0.975
SharinGAN [32]	N	0.116	0.939	5.068	0.203	0.850	0.948	0.978
SCSFM [15]	N	0.114	0.813	4.706	0.191	0.873	0.960	0.982
Monodepth2 [6]	N	0.112	0.851	4.754	0.190	0.881	0.960	0.981
SGDepth [8]	Seg	0.112	0.833	4.688	0.190	0.884	0.961	0.983
SAFENet [29]	Seg	0.112	0.788	4.582	0.187	0.878	0.963	0.983
PackNet-sfm [13]	N	0.111	0.785	4.601	0.189	0.878	0.960	0.982
HR-Depth [22]	N	0.109	0.792	4.632	0.185	0.884	0.962	0.983
Chanduri [33]	N	0.106	0.750	4.482	0.182	0.890	0.964	0.983
CADepth [34]	N	0.105	0.769	4.535	0.181	0.892	0.964	0.983
FeatDepth [28]	HR	0.104	0.729	4.481	0.179	0.893	0.965	0.984
GCNDepth [35]	HR	0.104	0.720	4.494	0.181	0.888	0.965	0.984
FSRE [21]	Seg	0.102	**0.675**	4.393	0.178	0.893	0.964	0.984
**Ours**	**Seg**	**0.101**	0.718	**4.376**	**0.176**	**0.896**	**0.966**	**0.984**

↓ (smaller values preferred) and ↑ (larger values preferred).

**Table 2:** Quantitative results of depth estimation on Make3D dataset for distance within 80 m.

Method	Sup	AbsRel ↓	SqRel ↓	RMSE ↓	RMSE_log ↓
Liu et al. [36]	Y	0.462	6.625	9.972	0.161
Laina et al. [9]	Y	0.204	1.840	5.683	0.084
Godard et al. [37]	Y	0.443	7.112	8.860	0.142
Zhou et al. [4]	N	0.392	4.473	8.307	0.194
DDVO [38]	N	0.387	4.720	8.090	0.204
Monodepth2 [6]	N	0.344	4.065	7.920	0.197
**Ours**	**N**	**0.337**	**3.842**	**7.733**	**0.190**

↓ (smaller values preferred) and ↑ (larger values preferred).

**Table 3:** Ablation study on KITTI dataset.

Method	AbsRel ↓	SqRel ↓	RMSE ↓	RMSE_log ↓	*a*_1_ ↑	*a*_2_ ↑	*a*_3_ ↑
w/o MSFAN, w/o Seg, w/o PSDIFM	0.109	0.792	4.632	0.185	0.884	0.962	0.983
+MSFAN	0.107	0.776	4.620	0.185	0.886	0.962	0.983
+MSFAN+Seg	0.107	0.766	4.511	0.183	0.892	0.965	0.983
+MAFAN+Seg+ PSDIFM	0.103	0.747	4.405	0.177	0.895	0.966	0.984
+MAFAN+Seg+ PSDIFM+Metric Loss	**0.101**	**0.718**	**4.376**	**0.176**	**0.896**	**0.966**	**0.984**

↓ (smaller values preferred) and ↑ (larger values preferred).

## Data Availability

The experimental data for this paper comes from two publicly available datasets, KITTI and Make3D. They are available at the following links respectively: 10.1109/CVPR.2012.6248074 and 10.1109/TPAMI.2008.132.
